# Subzero Control of Human Norovirus and Indicator Microorganisms on Frozen Foods Using an Antifreeze–Peracetic Acid Formulation

**DOI:** 10.3390/v18080846

**Published:** 2026-08-01

**Authors:** Yang Jiao, Zilei Zhang, Junshan Gao, Yuanling Chen, Yinghuan Xu, Danlei Liu, Musfirah Zulkurnain, Liang Xue

**Affiliations:** 1 Food Technology Division, School of Industrial Technology, Universiti Sains Malaysia (USM), Penang 11800, Malaysia; jiaoyang1996@student.usm.my; 2 State Key Laboratory of Applied Microbiology Southern China, Guangdong Provincial Key Laboratory of Microbial Safety and Health, National Health Commission Science and Technology Innovation Platform for Nutrition and Safety of Microbial Food, Key Laboratory of Big Data Technologies for Food Microbiological Safety, State Administration for Market Regulation, Institute of Microbiology, Guangdong Academy of Sciences, Guangzhou 510070, Chinachenyuanling20@163.com (Y.C.);; 3 Food and Drug Laboratory, Guangdong Detection Center of Microbiology, Guangzhou 510070, China; 4 Department of Customs Inspection and Quarantine, Shanghai Customs University, Shanghai 201204, China; 5 Shanghai International Travel Healthcare Center, Shanghai Customs District P. R. China, Shanghai 200335, China

**Keywords:** antifreeze, peracetic acid, frozen foods, subzero disinfection, norovirus, indicator microorganisms

## Abstract

Human norovirus (HuNoV) is one of the most important foodborne viruses and poses a significant risk in frozen foods along the cold chain. Traditional disinfectants exhibit reduced efficacy in frozen matrices and often fail to meet the requirements for food applications. This study optimized and evaluated a novel antifreeze–peracetic acid formulation (Af-PAA) for application on frozen foods, achieving disinfection performance at −40 °C. The formulation was optimized using four indicator microorganisms, with effective Af-PAA concentrations of 200 mg/L for *Escherichia coli*, 200 mg/L for *Staphylococcus aureus*, 500 mg/L for *Candida albicans*, and 2000 mg/L for *Aspergillus niger*. HuNoV GII.4[P31] propagated in zebrafish was inoculated onto frozen food samples. The frozen blueberries (500 mg/L), carrots (500 mg/L), chicken breast (2000 mg/L), and Arctic shrimp (2000 mg/L) were then subjected to Af-PAA treatment at −40 °C for 60 min with 60 s vortex mixing. RNase-RT-qPCR analysis showed a 3.0–3.1 log_10_ reduction in RNase-resistant HuNoV RNA signals across all tested food under the corresponding treatment concentrations. No visual changes were observed in treated foods. This study demonstrated that Af-PAA met tested food disinfectant requirements and effectively inactivated indicator microorganisms. HuNoV signals were also markedly reduced on the tested foods. These findings support its application for microbial risk control in cold-chain foods.

## 1. Introduction

Human norovirus (HuNoV) frequently contaminates frozen fruits, vegetables, meat, and seafood, posing an increasing risk to food safety worldwide [1]. Although refrigeration and freezing slow microbial growth, they can also prolong the survival of foodborne pathogens on food [2,3]. Viral contamination is frequently detected in imported and exported cold-chain foods and on their packaging surfaces [4]. This poses foodborne risks and increases cross-contamination to food handlers and consumers [5,6]. In addition, during thawing and handling, pathogens can be transferred to hands and food-contact surfaces [7,8,9]. Current sanitation practices mainly target packaging, equipment, and facility surfaces. Common disinfection approaches include spraying, fogging, ultraviolet (UV) irradiation, and cold plasma treatment. However, effective decontamination of frozen foods remains difficult under subzero conditions. The complex surface characteristics of foods and the presence of organic matter can hinder disinfectant contact with microorganisms, thereby reducing disinfection efficiency [10,11].

Most food disinfectants are developed for ambient or chilled conditions. They are widely used for fresh produce washing [12,13,14]. Only a few formulations have been evaluated under subzero conditions [15]. This highlights the need for food-grade disinfectants suitable for frozen foods. Such approaches can control contamination while reducing excessive chemical use [16,17]. Peracetic acid (PAA) is a broad-spectrum oxidizing disinfectant with a low environmental impact [18,19,20,21,22,23,24,25]. In 2025, China’s new national food safety standards approved the use of PAA for food disinfection, permitting concentrations up to 2000 mg/L [26]. Similar applications are also permitted by the USA FSIS [27]. These properties indicate that PAA-based formulations are promising candidates for disinfection of foods at subzero temperatures. Although PAA remains effective at −20 °C, its application requires an antifreeze system to ensure fluidity and adequate surface contact. Alcohol-based PAA formulations have demonstrated superior efficacy on frozen surfaces compared with chlorine-based disinfectants and quaternary ammonium compounds [15]. Several alcohols, including ethanol and isopropanol, have been investigated as antifreeze agents or food disinfectants for low-temperature applications [26,28,29,30,31,32].

HuNoV GII.4 [P31] is the dominant genotype responsible for most HuNoV outbreaks worldwide and is recognized for its persistence and resistance to disinfection [33]. Previous work has shown that HuNoV efficiently replicates in zebrafish larvae and embryos, providing a robust in vivo model for virus propagation and virology studies [34]. Most disinfection studies have relied on surrogate viruses or RT-qPCR detection of HuNoV from clinical samples. However, conventional RT-qPCR may detect residual viral RNA even after particle damage, which can complicate the interpretation of virus reduction in disinfection studies [35]. In this study, zebrafish-propagated HuNoV was used, and RNase pretreatment was combined with RT-qPCR to reduce signals from free viral RNA and severely damaged viral particles. This approach improves the interpretation of HuNoV genome-copy data compared with RT-qPCR alone, although the results should be interpreted as reductions in RNase-resistant HuNoV RNA signals rather than direct evidence of infectivity loss.

Disinfection of frozen foods without thawing is essential for maintaining cold-chain integrity and food quality. Products that can reliably disinfect frozen foods in real cold-chain environments remain scarce [36,37]. In recent years, human viruses have been repeatedly detected on imported frozen foods [38,39]. Therefore, there is an urgent need to develop effective disinfectant formulations and to evaluate their virucidal efficacy on frozen foods under subzero conditions. In this context, blueberries, carrots, Arctic shrimp, and chicken breast were selected as representative frozen foods commonly encountered in cold-chain systems. These food matrices vary in surface characteristics and composition, which can influence disinfection efficacy.

This study was designed to develop an antifreeze–peracetic acid (Af-PAA) formulation suitable for food disinfection under subzero conditions. The formulation was first optimized to meet established national disinfection standards and allow effective application on frozen food. Frozen foods were experimentally inoculated with infectious HuNoV propagated in zebrafish. The effect of Af-PAA on HuNoV levels on food was then evaluated under subzero conditions using RNase-RT-qPCR. The findings provide practical guidance for developing and applying food-grade disinfection strategies in cold-chain environments.

## 2. Materials and Methods

### 2.1. Preparation of Indicator Microorganisms and HuNoV Suspensions

*Escherichia coli* ATCC 8099 (*E. coli*), *Staphylococcus aureus* ATCC 6583 (*S. aureus*), *Candida albicans* ATCC 10231 (*C. albicans*), and *Aspergillus niger* ATCC 16404 (*A. niger*) were obtained from the Guangdong Culture Collection Center of Microorganisms (Guangzhou, China). Working cultures were prepared by routine subculturing. Bacterial strains were cultured on nutrient agar (NA) (Huankai Microbial, Guangzhou, China) or tryptic soy agar (TSA) (Guangzhou, China) at 37 °C, while C. albicans and A. niger were cultured on Sabouraud dextrose agar (SDA) (Huankai Microbial, Guangzhou, China) and malt extract agar (MEA) (Huankai Microbial, Guangzhou, China), respectively. Microbial suspensions were prepared in 0.03 mol·L^−1^ phosphate-buffered saline (PBS) (Huankai Microbial, Guangzhou, China) and adjusted to approximately 10^9^ CFU/mL.

HuNoV GII.4 [P31] was provided by the research group of Professor Xiaoxia Kou, Guangdong Medical University, China. The HuNoV strain used in this study was originally obtained from a clinical fecal sample, sequenced, and genotyped using the web-based Human Calicivirus Typing Tool (https://calicivirustypingtool.cdc.gov/, accessed on 1 July 2026). The RdRp and VP1 regions were assigned as P31 and GII.4, respectively, resulting in the final genotype GII.4[P31]. The FASTA sequence is provided in the Supplementary Materials. The virus was propagated in zebrafish and used as a replication-competent infectious virus suspension with a concentration of approximately 7 log_10_ genome copies/mL [40].

### 2.2. Sample Preparation and Inoculation

304 stainless steel coupons (12 mm diameter) were used. Blueberries, carrots, Arctic shrimp, and chicken breast were obtained from local retail markets in Guangzhou, China. Blueberries (intact, ~2.5 g, ~1.8 cm dia.), carrot cylinders (~2 cm length, ~4 g), chicken breast cuboids (2 × 2 × 1 cm, ~5 g), and Arctic shrimp (intact, ~5 cm, ~4.5 g) were prepared as test portions (*n* = 3 per treatment) [41,42]. Estimated surface areas were 10, 9, 16, and 25 cm^2^, respectively. Each sample was immersed in 20 mL Af-PAA solution, yielding disinfectant-to-sample ratios of 8.0, 5.0, 4.0, and 4.4 mL/g, respectively. A 100 μL aliquot of microbial suspension was applied onto the exposed surface area of each food sample using a micropipette. The inoculum was evenly distributed by depositing small droplets at multiple locations on the sample surface. A 100 μL aliquot of the inoculum was dispensed as multiple droplets onto the exposed surface of each food sample using a micropipette, covering approximately 50–70% of the surface area.

### 2.3. Antifreeze Formulation Optimization

Isopropanol was tested at concentrations of 0, 10, 20, 30, 40, 50, 60, 70 and 80% (*v*/*v*). Ethanol (95%) was tested at 0, 10, 20, 30, 40, 50, 60, and 70% (*v*/*v*). NaCl was added at 0, 0.5, 1, and 1.5% (*w*/*v*). Sterile standard hard water was used as the solvent.

Each formulation was adjusted to a final volume of 20 mL in a 50 mL centrifuge tube and mixed thoroughly. Formulations were stored at −20 °C and −40 °C for at least 8 h. After removal, the physical state was observed and recorded within 30 min. Each test was repeated 3 times. Antifreeze formulations were classified as frozen, showing salt phase separation (salting-out), or remaining as a homogeneous solution.

### 2.4. PAA Concentration Determination

#### 2.4.1. Preparation of Af-PAA and Neutralizer

The commercial stock solution contained approximately 20% hydrogen peroxide and 10% acetic acid. The active PAA concentration of the stock solution was 190 g/L, and all PAA concentrations reported in this study refer to active PAA. PAA solutions were prepared from a commercial 190 g/L stock solution (Huankai Microbial, Guangzhou, China) using sterile standard hard water. Af-PAA formulations were prepared by replacing water with the antifreeze carrier. The neutralizer consisted of 0.03 mol/L PBS supplemented with 1% polysorbate 80, 1% lecithin, and 0.5% sodium thiosulfate [32]. Neutralizer validation was conducted before all disinfection assays, including both microbial and viral tests, with the corresponding results reported in Supplementary Tables S1 and S2, respectively.

#### 2.4.2. Suspension and Stainless-Steel Coupon Tests

PAA concentrations of 25, 50, 100, 200, 500, 1000, and 2000 mg/L were evaluated in both suspension and stainless-steel coupon tests. Microbial suspensions were mixed with 3% bovine serum albumin (1:1, *v*/*v*) to simulate organic contamination. Viable bacteria were quantified by pour-plating 1.0 mL of the undiluted (10^0^) and serially diluted samples (ten-fold dilutions prepared by transferring 100 μL into 900 μL PBS), with a detection limit of 1 CFU/mL based on the 1.0 mL plated volume.

For suspension tests, microbial inocula (~1 × 10^7^–5 × 10^7^ CFU/mL) were exposed to Af-PAA for 10 min, neutralized with 10 mL neutralizer for 10 min, and then pour-plated as described above using dilutions up to 10^−5^. Untreated positive controls received sterile hard water instead of Af-PAA, and sterile PBS served as the negative control; both underwent the same neutralization and enumeration procedures.

For carrier tests, three contaminated stainless-steel coupons (~1 × 10^6^–5 × 10^6^ CFU/coupon) were immersed in 15 mL disinfectant for 10 min, then individually transferred into 5.0 mL neutralizer and neutralized for 10 min. The recovered suspensions were pour-plated as above using dilutions up to 10^−4^. The mean count of the three coupons represented one experimental result, and the entire experiment was independently repeated three times.

When no colonies were detected in the undiluted samples, the microbial level was recorded as below the detection limit. The detection limit was 1 CFU/mL for suspension tests and 5 CFU/coupon for coupon tests. Reductions were expressed as minimum confirmed reductions using “≥x log_10_ reduction.”

### 2.5. Temperature-Dependent Disinfection of Indicator Microorganisms

#### 2.5.1. Optimization on Stainless Steel

Af-PAA concentrations of 0, 100, and 200 mg/L for *E. coli* and *S. aureus*, 0, 200, and 500 mg/L for *C. albicans*, and 0, 1000, and 2000 mg/L for *A. niger* spores were tested. Solutions were equilibrated at room temperature (RT), 4 °C, −20 °C, and −40 °C. Stainless-steel coupons inoculated with indicator microorganisms were exposed to Af-PAA at the target temperature for the prescribed contact time of 10 min. Prior to treatment, both Af-PAA solutions and contaminated samples were pre-equilibrated at the designated test temperature.

#### 2.5.2. Optimization of Foods with *E. coli*

*E. coli* was used as the representative indicator organism for optimization at −40 °C. Af-PAA solutions at 100, 200, 500, 1000, and 2000 mg/L were prepared in the antifreeze base. Frozen blueberries and carrots were immersed in 20 mL of Af-PAA solution for 10 min and subjected to vortex mixing for 20, 40, or 60 s.

Chicken breast and Arctic shrimp were treated with Af-PAA for 10, 30, or 60 min, followed by vortex mixing for 60 s. After treatment, samples were transferred into neutralizer for quenching and microbial enumeration. Subsequently, the sample was neutralized with 10 mL of neutralizer for 10 min.

#### 2.5.3. Determining the Time-Concentration Relationship on Foods

At −40 °C, 20 mL of Af-PAA was used for each food sample, and vortex mixing for 20 or 60 s was applied as part of the disinfection procedure. For carrots and blueberries, Af-PAA was tested at 200 mg/L for *E. coli* and *S. aureus*, 500 mg/L for *C. albicans*, and 2000 mg/L for *A. niger* spores, with contact times of 5, 10, and 15 min. For chicken breast and Arctic shrimp, Af-PAA was applied at 2000 mg/L for all four indicator organisms, with contact times of 30, 60, and 90 min.

### 2.6. Af-PAA Inactivation of HuNoV on Frozen Foods

#### 2.6.1. Virus Recovery and Quantification

Virus recovery and concentration were performed using a modified ISO 15216-1:2017 method for food surfaces [43]. Briefly, recovered virus suspensions were concentrated using PEG/NaCl precipitation (Macklin, Shanghai, China) at 4 °C for 2 h, followed by centrifugation. The resulting pellet was resuspended in PBS and treated with RNase One (Promega, Madison, WI, USA) at 37 °C for 15 min to remove exposed viral RNA [44]. Viral RNA was extracted using a Viral RNA Extraction Kit (Huankai Microbial, China).

HuNoV was quantified using a QuantStudio 5 Real-Time PCR System (Thermo Fisher Scientific, Waltham, MA, USA) and an RNA Probe One-Step qRT-PCR Kit (Huankai Microbial, Guangzhou, China). The primers and probe used for NoV GII detection were QNIF2d (5′ ATG TTC AGR TGG ATG AGR TTC TCW GA 3′), COG2R (5′ TCG ACG CCA TCT TCA TTC ACA 3′), and QNIFS (5′ FAM-AGC ACG TGG GAG GGG ATC G-TAMRA) for NoV GII [45]. The HuNoV primer set generated an 89 bp amplicon. Thermal cycling conditions were as follows: reverse transcription at 42 °C for 300 s, inactivation of reverse transcriptase at 95 °C for 10 min, followed by 45 cycles of denaturation at 95 °C for 5 s and annealing at 60 °C for 20 s.

The analytical performance of the HuNoV RNase-RT-qPCR assays was evaluated based on the standard curve parameters, including slope, intercept, R^2^, and amplification efficiency. Across the five independent standard curves, the slopes ranged from −3.416 to −3.237, with R^2^ values of 0.998–0.999 and amplification efficiencies of 96.2–103.7% in Supplementary Table S3. MNV was used as a post-neutralization analytical recovery control, and the corresponding recovery results are summarized in Supplementary Table S4. Matrix-specific RT-qPCR inhibition was assessed using extracted HuNoV RNA, and the corresponding results are provided in Supplementary Table S5. The practical LOD was 10 genome copies/μL, and an estimated quantification threshold of 40 genome copies/μL was conservatively defined as four times the practical LOD [46].

#### 2.6.2. Virucidal Assay on Frozen Foods

Frozen blueberries, carrots, chicken breast, and Arctic shrimp were surface-inoculated with 100 μL of HuNoV suspension (~10^7^ genome copies/mL). After inoculation, the foods were equilibrated at −40 °C prior to treatment with Af-PAA for 30, 60, or 90 min at concentrations of blueberries and carrots (500 mg/L) and chicken breast and Arctic shrimp (2000 mg/L). After inoculation, samples were immersed in 20 mL of Af-PAA solution and vortex-mixed for 60 s during the disinfection treatment. Following treatment, viruses were recovered and neutralized with 10 mL of neutralizer for 10 min. Low-temperature stability controls were included to evaluate the effect of frozen exposure alone. HuNoV-contaminated food matrices were held at −40 °C for 30, 60, and 90 min without disinfectant treatment and analyzed by RNase-RT-qPCR.

### 2.7. Visual Quality Assessment

Visual changes in the foods were evaluated at RT by comparing untreated controls with Af-PAA-treated samples, including blueberry, carrot, chicken breast, and Arctic shrimp. After disinfection, samples were rinsed with sterile water and allowed to air-dry under aseptic conditions prior to assessment.

### 2.8. Statistical Analysis

Viable counts were determined by the pour-plate method, and plates containing 30–300 CFU were used for calculation. Microbial counts were converted to log_10_ CFU values before statistical analysis. Log reduction (LR) was calculated as LR = log_10_N_0_ − log_10_N_t_, where N_0_ represents the corresponding untreated control, and N_t_ represents the treated sample.

For HuNoV, viral genome copy numbers obtained by RNase-RT-qPCR were calculated from the corresponding standard curves and converted to log_10_ genome copies/μL. HuNoV reductions were calculated relative to the corresponding positive controls.

All experiments were performed in triplicate, and data are presented as mean ± SD. Statistical analyses were performed using GraphPad Prism 10.1.2 and Microsoft Excel 2021. For comparisons among multiple treatment groups, one-way analysis of variance (one-way ANOVA) followed by Tukey’s multiple-comparison test was used. When two experimental factors were analyzed, a two-way ANOVA followed by Tukey’s multiple-comparison test was performed. A value of *p* < 0.05 was considered statistically significant. Statistical significance is indicated in the corresponding figures and figure captions.

## 3. Results

### 3.1. Optimization and Characterization of the Antifreeze

**Figure 1:**
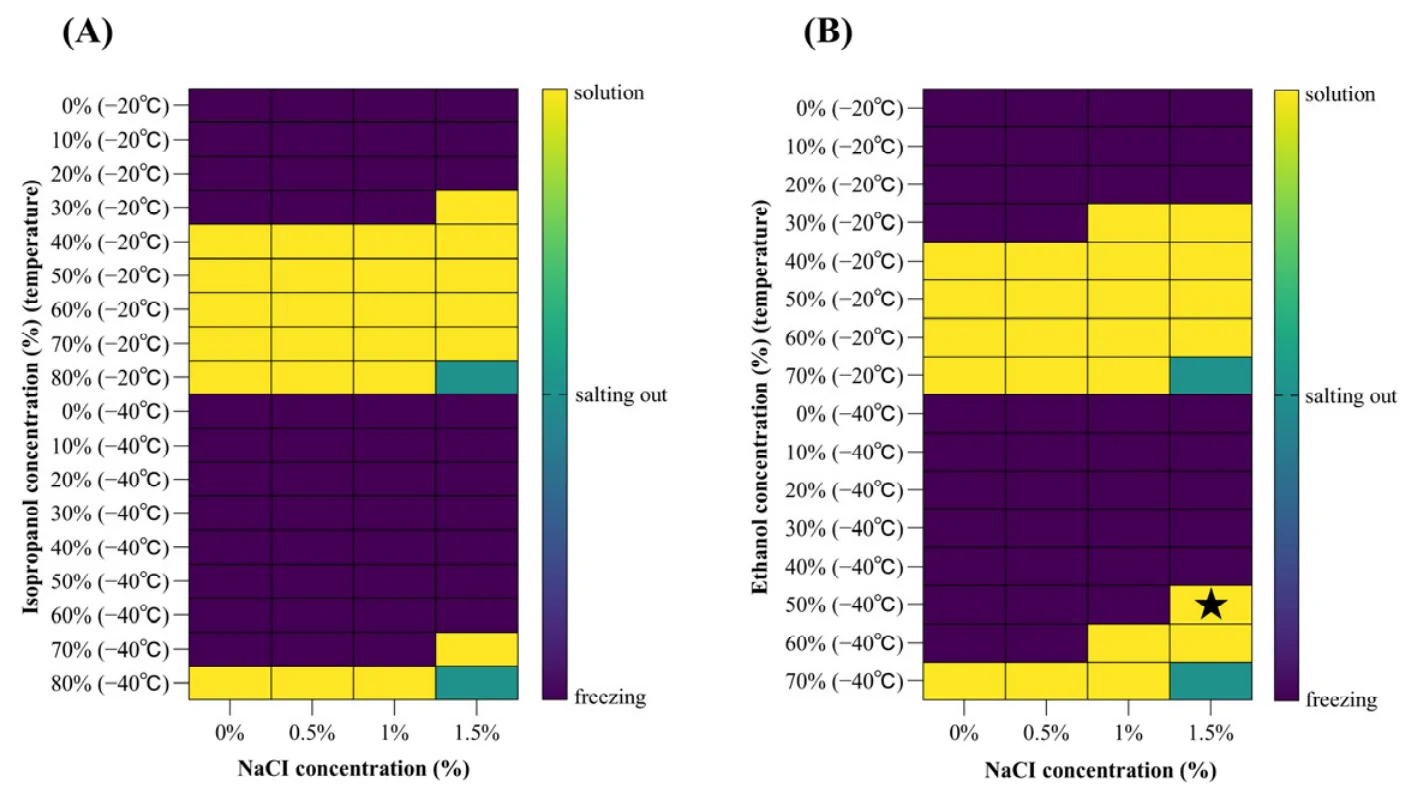
Physical states of isopropanol–NaCl (**A**) and ethanol–NaCl (**B**) formulations at −20 °C and −40 °C. Each cell represents the observed phase after freeze–thaw equilibration (solution, salting out, or freezing). ★ indicates the optimal concentration within the solution region.

The antifreeze performance of ethanol–NaCl and isopropanol–NaCl systems was evaluated at −20 °C and −40 °C in Figure 1. The ethanol–NaCl system showed superior low-temperature stability, with 50% (*v*/*v*) ethanol and 1.5% (*w*/*v*) NaCl remaining as a clear, unfrozen single phase for at least 8 h at both temperatures. This formulation was therefore selected as the antifreeze base for subsequent Af-PAA preparation.

### 3.2. Effective PAA Concentrations at RT

**Figure 2:**
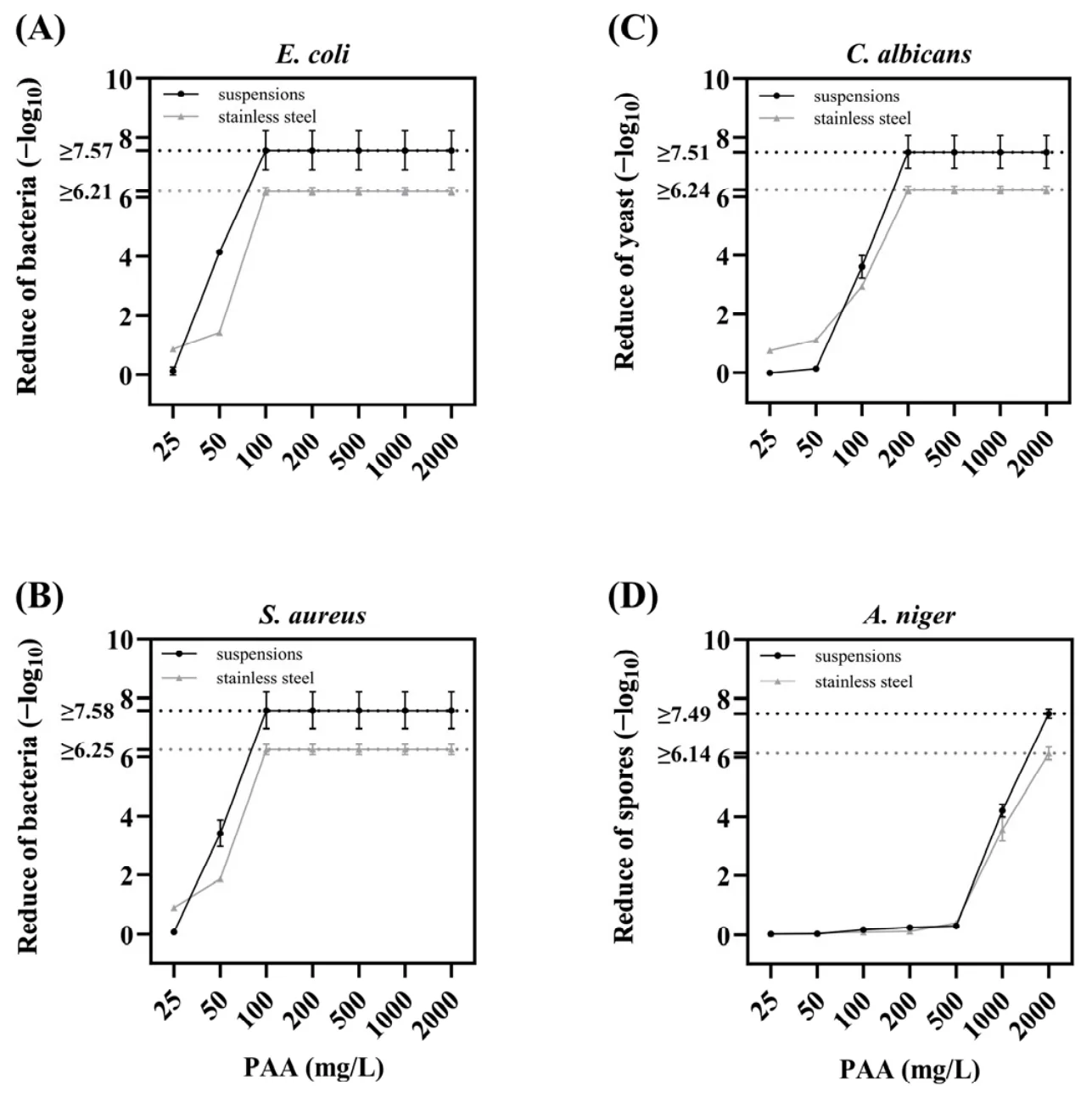
Concentration-dependent antimicrobial activity of PAA at RT against (**A**) *E. coli*, (**B**) *S. aureus*, (**C**) *C. albicans* and (**D**) *A. niger* spores in suspension assays and on stainless steel. Log_10_ reductions were determined after 10 min exposure at 25–2000 mg/L PAA. For treatment groups in which no colonies were detected, the plotted values represent minimum confirmed log_10_ reductions calculated using the microbiological detection limit of 1 CFU/mL and the corresponding untreated positive controls. The black and gray horizontal dotted lines indicate the minimum confirmed reductions for the suspension and stainless-steel coupon assays, respectively, and are denoted by “≥”.

PAA exhibited concentration-dependent antimicrobial activity against all indicator microorganisms in Figure 2. At 100 mg/L, viable *E. coli* and *S. aureus* were reduced to below the microbiological detection limit in both suspension and stainless-steel coupon assays. These non-detectable endpoints corresponded to minimum confirmed reductions of ≥7.57 and ≥7.51 log_10_ in suspension, and ≥6.21 and ≥6.24 log_10_ on stainless-steel coupons, respectively. *C. albicans* required 200 mg/L PAA to reach below-detection levels, corresponding to minimum confirmed reductions of ≥7.51 log_10_ in suspension and ≥6.24 log_10_ on stainless-steel coupons. A. niger spores were the most resistant and remained detectable at 1000 mg/L. Non-detectable levels were reached only at 2000 mg/L, corresponding to minimum confirmed reductions of ≥7.49 log_10_ in suspension and ≥6.14 log_10_ on stainless-steel coupons.

### 3.3. Temperature-Dependent Disinfection on Stainless Steel

**Figure 3:**
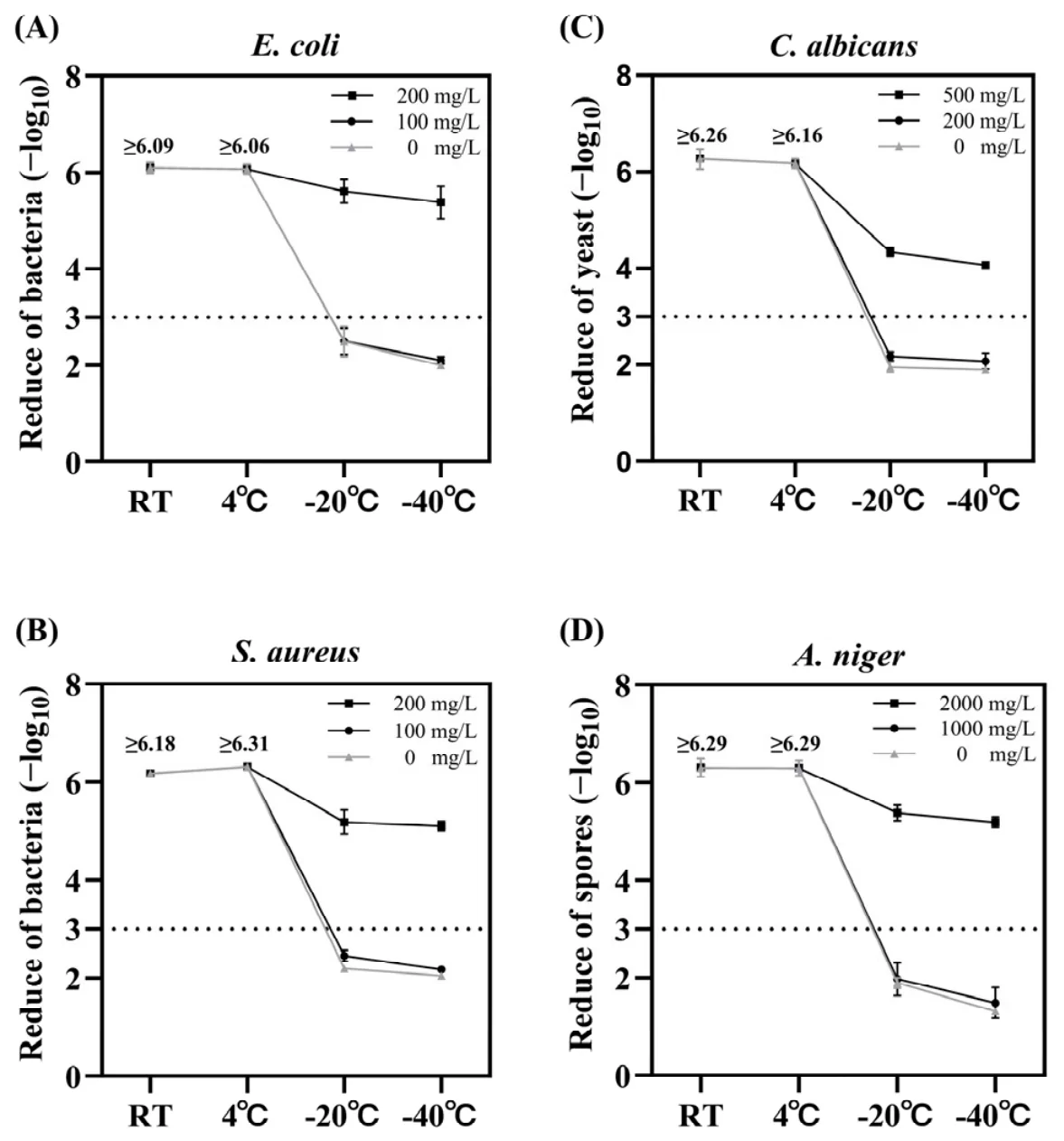
Reduction in *E. coli* (**A**), *S. aureus* (**B**), *C. albicans* (**C**) and *A. niger* spores (**D**) on stainless steel treated with Af-PAA at different temperatures. Stainless-steel coupons were exposed to Af-PAA solutions across a range of concentrations at RT, 4 °C, −20 °C, and −40 °C. Values marked with “≥” indicate that no colonies were detected and that the viable counts were below the microbiological detection limit of 1 CFU/mL; these values represent the minimum confirmed log_10_ reductions calculated from the corresponding untreated positive-control counts.

At RT and 4 °C, 100 mg/L Af-PAA achieved >5 log reductions in *E. coli* and *S. aureus*, while 200 mg/L was required for *C. albicans* and 1000 mg/L for *A. niger* spores in Figure 3. The PAA-free 50% ethanol–NaCl antifreeze carrier showed strong antimicrobial activity at RT and met the disinfection requirement for the tested indicator microorganisms, indicating that the antifreeze carrier itself had considerable disinfectant activity under above-freezing conditions. After Af-PAA treatment, comparable disinfection performance was also achieved at RT and 4 °C, with viable counts reduced to below the detection limit at the effective concentrations. Therefore, no significant difference in microbial reduction was observed between RT and 4 °C, as both treatments reached the same non-detectable endpoint. However, these concentrations became ineffective at −20 °C and −40 °C. Increasing the concentration to 200 mg/L for *E. coli* and *S. aureus*, 500 mg/L for *C. albicans*, and 2000 mg/L for *A. niger* spores restored effective disinfection under subzero conditions, achieving >3 log reductions on stainless steel. The antifreeze carrier alone showed limited antimicrobial activity.

### 3.4. Concentration Optimization on Frozen Foods

**Figure 4:**
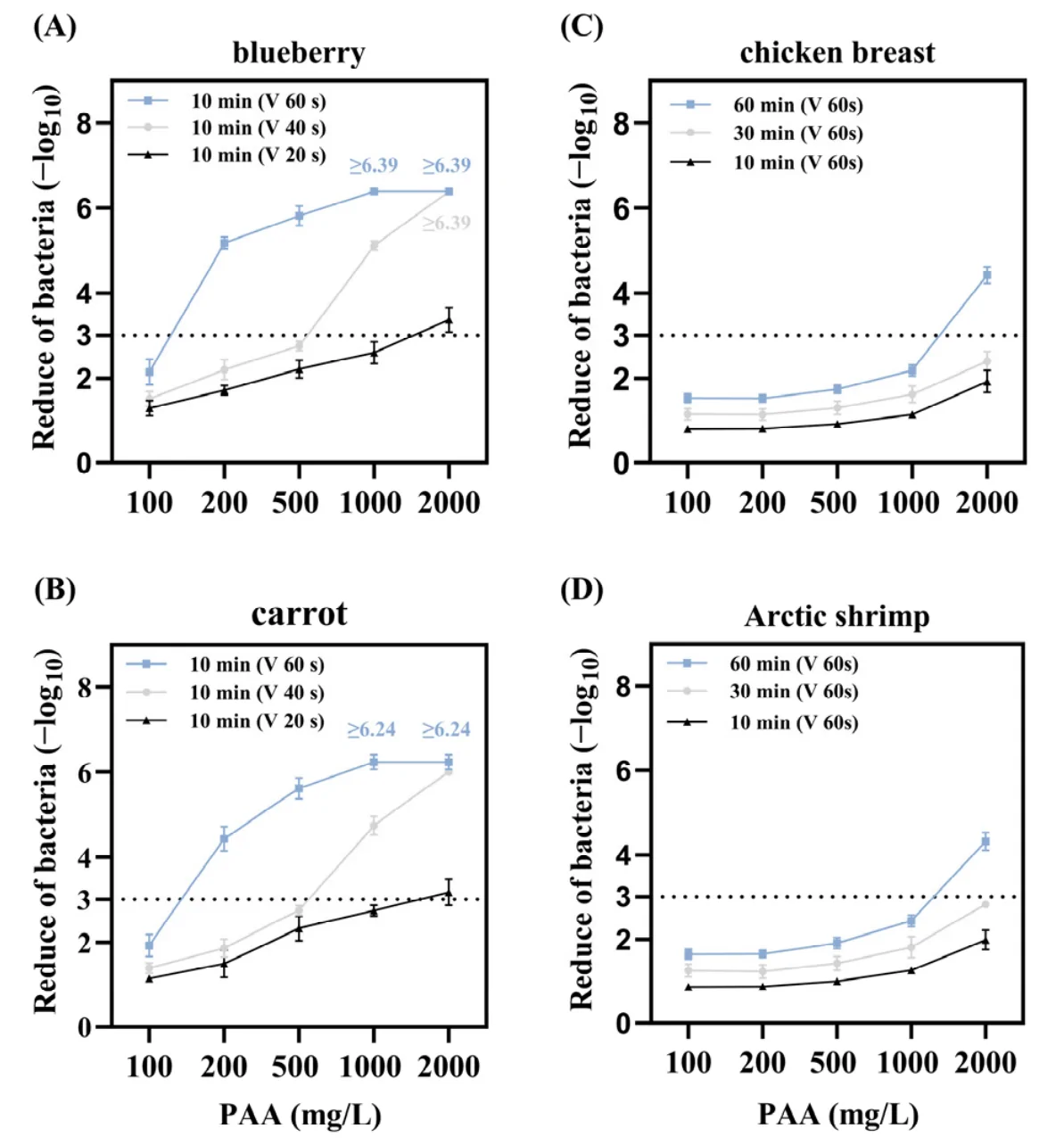
Concentration-dependent bacterial inactivation on frozen foods under subzero conditions using Af-PAA. Panels (**A**–**D**) show results for blueberry (**A**), carrot (**B**), chicken breast (**C**), and Arctic shrimp (**D**). Blueberry and carrot were treated for 10 min with vortex mixing (20–60 s), whereas chicken breast and Arctic shrimp were treated for longer durations (10–60 min) with 60 s vortex mixing. The dotted line indicates the 3 log_10_ reduction threshold for effective disinfection. Values marked with “≥” indicate that no colonies were detected and that the viable counts were below the microbiological detection limit of 1 CFU/mL; these values represent the minimum confirmed log_10_ reductions calculated from the corresponding untreated positive-control counts.

At −40 °C, concentration–time optimization was performed using *E. coli* as a representative indicator. Blueberries and carrots in Figure 4A,B received 100 mg/L Af-PAA for 10 min, which was insufficient for effective inactivation. Reductions above 3 log were achieved at 200 mg/L combined with 60 s vortex mixing. Higher concentrations from 500 to 1000 mg/L required vortex times of 40 s or longer to reach the same threshold.

Chicken breast and Arctic shrimp in Figure 4C,D treated with 100 to 1000 mg/L Af-PAA, even with 60 min contact and 60 s vortex mixing, did not reach 3 log reduction. Effective inactivation above 3 log was only obtained at 2000 mg/L with 60 min contact and 60 s vortex mixing.

### 3.5. Microbial Inactivation on Frozen Foods at −40 °C

**Figure 5:**
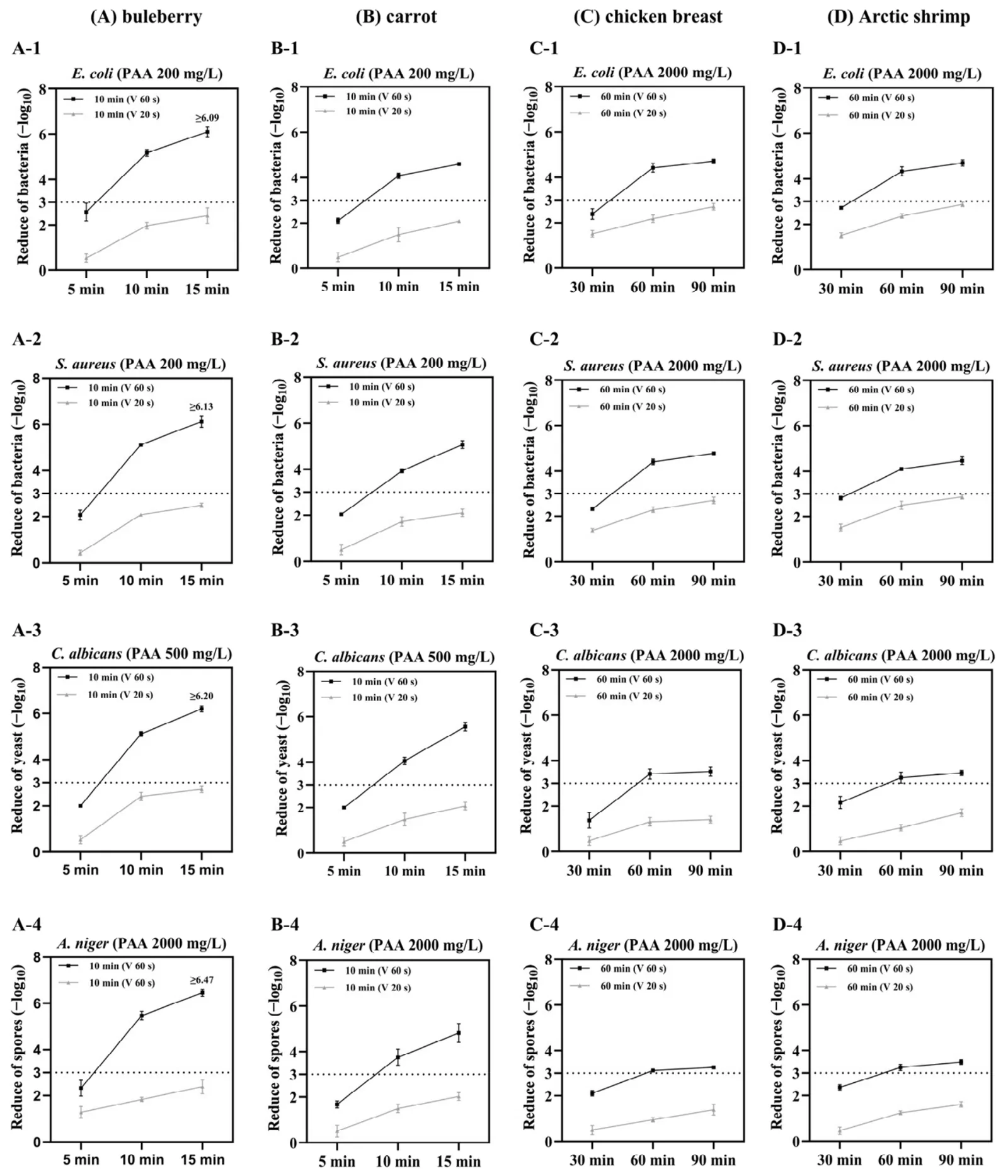
Time-dependent inactivation of indicator microorganisms on frozen foods under subzero conditions using Af-PAA. Panels (**A**–**D**) show microbial reductions on blueberry (**A1**–**A4**), carrot (**B1**–**B4**), chicken breast (**C1**–**C4**), and Arctic shrimp (**D1**–**D4**). In each panel, labels 1–4 represent *E. coli*, *S.aureus*, *C. albicans*, and *A.niger spores*, respectively. Different Af-PAA concentrations were applied depending on the food matrix. Treatments were conducted with vortex mixing (20 or 60 s) over time. The dotted line indicates the 3 log_10_ reduction threshold for effective disinfection. Values marked with “≥” indicate that no colonies were detected and that the viable counts were below the microbiological detection limit of 1 CFU/mL; these values represent the minimum confirmed log_10_ reductions calculated from the corresponding untreated positive-control counts.

On blueberries and carrots, microbial reductions increased with contact time and were consistently higher with 60 s vortex mixing than with 20 s (Figure 5A,B). Effective disinfection (>3 log reduction) was achieved after 10 min using 60 s vortex mixing for all four indicator microorganisms. Further extending the contact time to 15 min resulted in additional reduction, whereas 20 s vortex mixing remained less effective throughout the treatment period.

Similar trends were observed on chicken breast and Arctic shrimp (Figure 5C,D). Microbial reductions increased with contact time and were enhanced by 60 s vortex mixing. Effective disinfection was achieved after 60 min, and extending the contact time to 90 min provided only limited additional reduction. Under all tested conditions, 20 s vortex mixing failed to achieve the 3 log reduction criterion.

### 3.6. Norovirus Recovery from Foods at −40 °C

The recovery efficiency of HuNoV from different frozen food matrices is summarized in Table 1. Recovery rates were highest for blueberries (16.16%) and carrots (7.25%) and lower for chicken breast (5.49%) and Arctic shrimp (6.68%). The control sample was defined as the positive input virus control, which represented the initial HuNoV load before food matrix recovery and was set to 100% for recovery calculation. These results indicate that virus recovery from frozen food is limited and matrix-dependent. RNase pretreatment combined with PEG/NaCl concentration was used to improve virus recovery and reduce free RNA signals.

### 3.7. HuNoV Disinfection on Food at −40 °C

Before disinfection treatment, the stability of HuNoV on food matrices at −40 °C was evaluated. HuNoV levels remained stable on all tested food matrices during 30–90 min of frozen exposure, with SD values of 0.06–0.11 log_10_ genome copies/μL and a maximum mean change in only 0.05 log_10_ units (Supplementary Figure S1).

**Figure 6:**
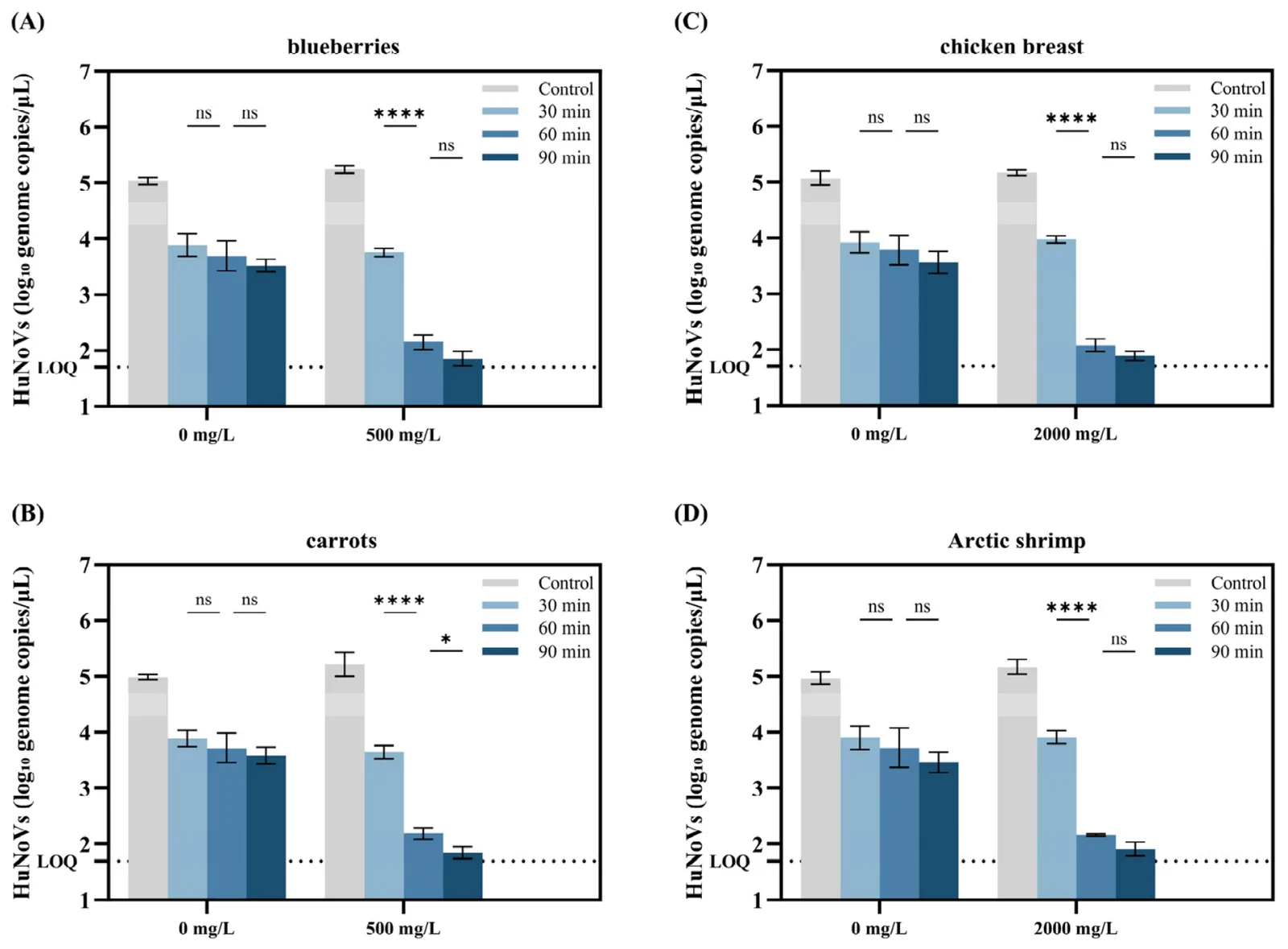
Disinfection performance of Af-PAA against HuNoV on different food matrices at −40 °C. HuNoV-contaminated food matrices were treated with the PAA-free antifreeze carrier or Af-PAA for 30, 60, and 90 min. The 0 mg/L group represents the antifreeze carrier without PAA. Af-PAA was applied at 500 mg/L for blueberries (**A**) and carrots (**B**), and at 2000 mg/L for chicken breast (**C**) and Arctic shrimp (**D**). HuNoV signals were quantified by RNase-RT-qPCR and expressed as log_10_ genome copies/μL. The dotted horizontal line indicates the estimated quantification threshold of 1.60 log_10_ genome copies/μL. For each food matrix, data obtained at 30, 60, and 90 min were analyzed using an ordinary two-way ANOVA, with Af-PAA concentration and treatment time as the two factors, followed by Tukey’s multiple-comparisons test comparing treatment times within each concentration level. The initial control values were displayed as reference values and were not included in the two-way ANOVA. The significance symbols shown indicate the 30 versus 60 min and 60 versus 90 min comparisons. Data are presented as mean ± SD (*n* = 3). ns, not significant; * *p* < 0.05; **** *p* < 0.0001.

The reduction in HuNoV levels on different food matrices at −40 °C is shown in Figure 6. In the 0 mg/L group, which represents the PAA-free antifreeze carrier, the mean initial HuNoV levels ranged from 4.97 to 5.07 log_10_ genome copies/μL across the four food matrices. After treatment with the antifreeze carrier alone, HuNoV levels decreased to 3.89–3.92 log_10_ genome copies/μL at 30 min, 3.69–3.79 log_10_ genome copies/μL at 60 min, and 3.47–3.59 log_10_ genome copies/μL at 90 min. These values corresponded to reductions of approximately 1.06–1.15, 1.24–1.34, and 1.40–1.52 log_10_ units after 30, 60, and 90 min, respectively.

In the Af-PAA treatment group, the mean initial HuNoV levels ranged from 5.17 to 5.24 log_10_ genome copies/μL. After 30 min of Af-PAA treatment, HuNoV levels decreased to 3.65–3.98 log_10_ genome copies/μL, corresponding to reductions of 1.19–1.57 log_10_ units. A greater reduction was observed after 60 min, with residual HuNoV levels declining to 2.08–2.19 log_10_ genome copies/μL, corresponding to reductions of 3.01–3.09 log_10_ units. Extending the treatment to 90 min further reduced HuNoV levels to 1.85–1.91 log_10_ genome copies/μL, corresponding to reductions of 3.26–3.38 log_10_ units. However, these residual levels approached the LOQ of the assay, and the additional reduction from 60 to 90 min was limited, ranging from only 0.18 to 0.35 log_10_ units.

Across all tested food matrices, Af-PAA treatment resulted in greater reductions in HuNoV levels than the PAA-free antifreeze carrier at −40 °C. Notably, the 60 min treatment achieved approximately 3.0 log_10_ reduction on all tested food matrices and therefore represented the key effective treatment time for HuNoV reduction under the tested frozen-food conditions.

### 3.8. Evaluation of Visual Quality Changes

**Figure 7:**
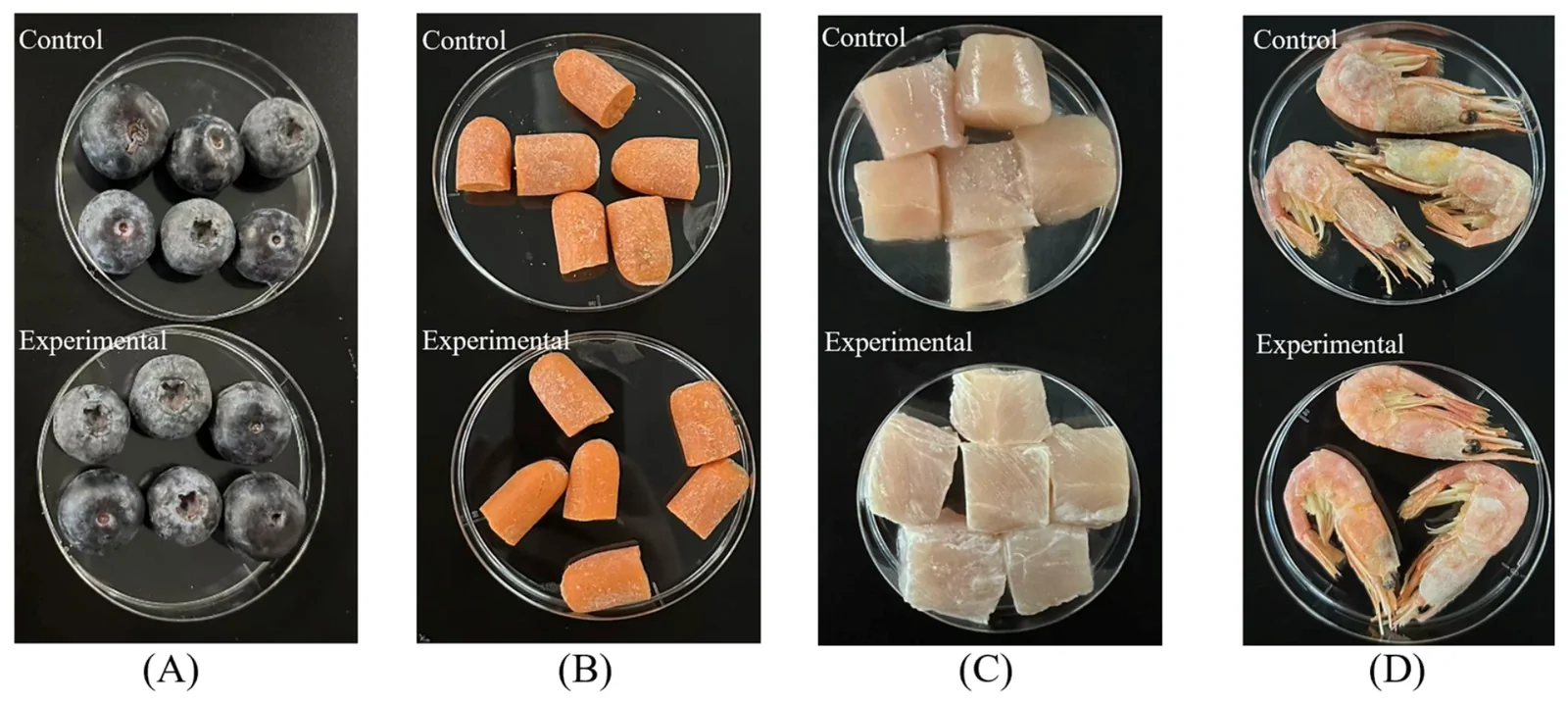
Representative images of blueberries (**A**), carrots (**B**), chicken breast (**C**), and Arctic shrimp (**D**) before and after Af-PAA treatment.

Representative images of untreated and Af-PAA-treated samples are shown in Figure 7. No obvious changes in color or surface condition were observed for blueberries (A), carrots (B), or Arctic shrimp (C) following treatment. Chicken breast (D) showed slight changes in surface condition.

## 4. Discussion

Disinfection of frozen foods and food-contact surfaces is challenging due to reduced fluidity of disinfectants and limited surface contact. Conventional water-based disinfectants often lose activity below 0 °C or fail to meet food-contact safety standards, and few studies have directly assessed efficacy under frozen conditions. The present study developed a food-grade antifreeze formulation combined with PAA that met standard disinfection criteria at RT. The formulation effectively inactivated indicator microorganisms under frozen conditions and was subsequently applied to artificially contaminated representative frozen foods. Both the indicator microorganisms and zebrafish-propagated HuNoV achieved reductions exceeding 3 log, demonstrating the potential of Af-PAA for effective microbial and viral control in cold-chain food environments.

Developing disinfectants for frozen food applications requires antifreeze components that remain compatible with the active biocide. In this study, ethanol and NaCl were combined with PAA to formulate a food-grade antifreeze disinfectant [26]. The selected formulation containing 50% ethanol and 1.5% NaCl remained fluid at −40 °C and retained antimicrobial activity under frozen conditions. The antifreeze matrix alone produced measurable reductions in indicator microorganisms, particularly at subzero temperatures. The antimicrobial efficacy of Af-PAA was evaluated according to the Chinese national standard GB 38502 [47]. At RT, effective PAA concentrations were 100 mg/L for *E. coli* and *S. aureus*, 200 mg/L for *C. albicans*, and 1000 mg/L for *A. niger* spores, achieving the required disinfection criteria in both suspension and stainless-steel coupon tests. These results established the baseline concentrations for subsequent low-temperature evaluations. A PAA-only aqueous control could not be performed at −40 °C because the solution froze under the test conditions. Therefore, the PAA-free antifreeze carrier was used as the carrier control and compared with Af-PAA to assess the additional contribution of PAA within the antifreeze formulation.

Disinfection efficacy was evaluated according to the Chinese national standard GB 38502 using both microbial suspensions and stainless steel coupons [47]. At RT, effective PAA concentrations were 100 mg/L for *E. coli* and *S. aureus*, 200 mg/L for *C. albicans*, and 1000 mg/L for *A. niger* spores. These levels achieved 4 log reductions in suspensions and 3 log reductions on stainless steel within 10 min. The concentration–time relationships align with reports showing that formulations containing 0.1% PAA produce 4 log reductions in bacteria and yeasts on environmental surfaces and stainless steel within 5 min at ambient temperature [48].

Optimizing Af-PAA concentrations is essential to ensure efficacy at subzero temperatures while avoiding overdosing and to protect consumer health [49,50]. At −40 °C, effective concentrations increased to 200 mg/L for *E. coli* and *S. aureus*, 500 mg/L for *C. albicans*, and 2000 mg/L for *A. niger* spores. This increase reflects reduced molecular diffusion, limited oxidant penetration, and restricted liquid–solid contact under freezing conditions. The lower susceptibility of *A. niger* spores to Af-PAA compared with bacteria and yeast may be attributed to the intrinsic structural resistance of fungal spores. PAA exerts antimicrobial activity mainly through oxidative damage to cellular membranes, cell walls, proteins, and other essential structures. Previous studies have reported that PAA-related inactivation of *A. niger* is associated with membrane damage and elevated ROS levels. Nevertheless, the robust protective structures of fungal spores may limit oxidant penetration and reduce disinfection efficiency, thereby requiring higher Af-PAA concentrations for effective inactivation [51].

Blueberries, carrots, chicken breast, and Arctic shrimp were selected as representative frozen foods [52,53,54,55]. Compared with stainless steel, these foods present additional challenges due to surface irregularities, organic residues, and rapid freezing, which impede disinfectant spreading [56]. The matrix-dependent efficacy of Af-PAA may be related to food composition and surface properties. Compared with blueberries and carrots, chicken breast and Arctic shrimp contain more proteins, lipids, and organic matter and have more complex surfaces, which may increase oxidant demand and limit disinfectant contact. Under subzero conditions, reduced reaction kinetics and mass transfer may further require longer contact times to achieve effective disinfection.

In this study, a short 20 s vortex step was sufficient for metal carriers but inadequate for frozen foods, indicating that physical contact—not chemical concentration—is the principal limiting factor at −40 °C. Extending vortexing to 60 s restored antimicrobial efficacy and allowed all frozen food matrices to reach the preset reduction targets. Time-response experiments further showed that extended surface contact improved microbial inactivation and led to lower residual HuNoV levels under frozen-food conditions. These results indicate that the required effective contact time was matrix-dependent. Fruit and vegetable matrices, including blueberries and carrots, showed effective reductions after more than 10 min of treatment, whereas animal-derived matrices, including chicken breast and Arctic shrimp, required more than 60 min. This suggests that complex animal-derived food matrices require prolonged Af-PAA contact to achieve the target disinfection effect.

Under these conditions, blueberries and carrots were effectively disinfected within 10 min using 200 mg/L Af-PAA for *E. coli* and *S. aureus* and 500 mg/L for *C. albicans*, whereas 2000 mg/L was required for *A. niger* spores. In contrast, chicken breast and Arctic shrimp required 2000 mg/L Af-PAA with 60 min contact time to achieve the target reductions for all tested microorganisms.

For HuNoV disinfection, oxidizing disinfectants such as peracetic acid in Af-PAA can disrupt viral capsid proteins, rendering viruses unable to bind to host cells. However, conventional RT-qPCR may still detect RNA from damaged viral particles, leading to potential overestimation of viral persistence [19]. To address this, the present study employed replication-competent HuNoV propagated in zebrafish and assessed HuNoV levels using RNase pretreatment prior to RT-qPCR [57]. This approach degrades free RNA from disrupted viral particles, allowing the quantification to better reflect RNase-resistant HuNoV RNA signals. Using this method, RNase-RT-qPCR analysis showed reductions of approximately 3.01–3.09 log_10_ on frozen food after 60 min of Af-PAA treatment, demonstrating effective viral control [58,59,60]. The 50% ethanol-containing antifreeze carrier itself contributed to HuNoV reduction, producing approximately 1.06–1.52 log_10_ reductions at −40 °C. However, Af-PAA achieved greater reductions than the carrier-only group, indicating that the observed effect resulted from the combined action of the ethanol-containing carrier and PAA. Notably, this level of viral reduction was achieved under −40 °C conditions and exceeded that reported for conventional thermal treatment at 80 °C for 10 min [61]. These results indicate that disinfection conditions effective against standard indicator microorganisms also achieved marked reductions in HuNoV levels detected by RNase-RT-qPCR under subzero conditions. Minimal differences in virus inactivation were observed among blueberries, carrots, chicken breast, and Arctic shrimp, highlighting the broad applicability of Af-PAA for controlling viral contamination in frozen food systems.

A limitation of this study is that HuNoV was evaluated using RNase pretreatment combined with RT-qPCR. Although RNase pretreatment reduces signals from free RNA and severely damaged viral particles, it cannot directly determine viral infectivity. Therefore, the observed reductions should be interpreted as decreases in RNase-resistant HuNoV RNA signals rather than direct evidence of infectious virus inactivation.

From an application perspective, no obvious visual changes were observed in blueberries, carrots, or Arctic shrimp after Af-PAA treatment. Chicken breast exhibited slight surface whitening. Previous studies have reported that such surface bleaching caused by PAA does not significantly affect cooking loss or processing performance [62].

The disinfection performance of Af-PAA showed clear matrix dependence. Arctic shrimp represents a complex seafood matrix with a high organic load and irregular surface structure, which may reduce effective disinfectant contact and reaction efficiency. This issue is particularly relevant to regulatory applicability because the permitted or notified use levels of PAA differ substantially among food categories and application scenarios. For example, FDA FCN No. 2352 lists maximum PAA levels of 2000 ppm for meat and poultry carcasses, parts, trim, and organs; 600 ppm for fruit and vegetable processing water and ice; and 230 ppm for fish and seafood processing water and ice [63]. Therefore, the 2000 mg/L PAA treatment used for Arctic shrimp in this study should be interpreted as an experimental evaluation under a challenging seafood matrix rather than as a directly applicable regulatory use level for seafood processing. Further optimization within the seafood-relevant PAA range is still needed. In addition, ethanol was selected as the main antifreeze component because it has established food-related uses and is listed by the FDA as a food-related substance with technical effects, including antimicrobial agent, processing aid, and solvent or vehicle [64].

The use of stainless steel and selected frozen foods under controlled laboratory conditions cannot fully reproduce the structural complexity, organic soiling, or operational variability of industrial processing lines. Therefore, this study should be regarded as a laboratory-scale proof of concept. Further studies are required to evaluate chemical residues, sensory attributes, product quality, process compatibility, and safety and to validate the treatment at pilot and industrial scales before practical application. In addition, although four representative frozen foods were examined, commodities with markedly different surface morphologies or fat contents may respond differently to Af-PAA application. Future work should assess residual oxidant behavior at subzero temperatures and the effectiveness of spraying or misting systems that may further improve surface coverage under frozen conditions.

## 5. Conclusions

This study developed an Af-PAA disinfectant for application on frozen foods under subzero conditions. The formulation remained fluid at low temperatures and effectively inactivated indicator microorganisms on stainless steel and representative frozen food matrices. Importantly, Af-PAA markedly reduced RNase-resistant HuNoV RNA levels on frozen foods at −40 °C, suggesting its potential application for reducing foodborne viral contamination under subzero conditions. Blueberries and carrots required lower treatment intensities than chicken breast and Arctic shrimp, highlighting the influence of the food matrix on disinfection efficacy. These results provide practical concentration–time guidance for cold-chain disinfection and support the application of Af-PAA for controlling both microbial and viral contamination in frozen food systems.

## Figures and Tables

**Table 1:** Recovery of HuNoV from different frozen food matrices.

Sample	Mean ± SD (log_10_ Genome Copies)	Recovery (%)
Control	5.766	100
Blueberries	4.933 ± 0.193	16.16 (9.42–22.91)
Carrots	4.624 ± 0.044	7.25 (6.52–7.98)
Chicken breast	4.475 ± 0.164	5.49 (3.51–7.46)
Arctic shrimp	4.556 ± 0.176	6.68 (4.11–9.25)

Note: Recovery (%) was calculated relative to the input control group (set as 100%). HuNoV recovery was evaluated from different frozen food matrices following surface inoculation and recovery procedures. HuNoV concentrations were determined by RT-qPCR and expressed as log_10_ genome copies/μL. Data are presented as mean ± standard deviation (SD), *n* = 3 independent experiments. Recovery efficiency was calculated using the following equation: $\mathrm{Recovery}(\%)\;=10^{\left(\log_{10}N_{\mathrm{sample}}-\log_{10}N_{\mathrm{control}}\right)}\times 100$ where $\log_{10}N_{\mathrm{sample}}\;$ represents the mean log_10_ HuNoV concentration recovered from each food matrix and $\log_{10}N_{\mathrm{control}}$ represents the mean log_10_ HuNoV concentration of the input control group.

## Data Availability

The raw data supporting the findings of this study, including the original Cq values generated from RT-qPCR assays and the experimental datasets, are available as separate data files provided with the submission.

## Supplementary Materials

The following supporting information can be downloaded at https://www.mdpi.com/article/10.3390/v18080846/s1; Table S1. Identification of neutralizing agents for disinfectants; Table S2. Neutralizer validation for HuNoV RT-qPCR; Table S3. Performance characteristics of the HuNoV RT-qPCR assay; Table S4. MNV process control recovery after neutralization; Table S5. RT-qPCR inhibition assessment in different food matrix extracts; Figure S1. HuNoV-contaminated blueberries, carrots, chicken breast, and Arctic shrimp were held at −40 °C for 30, 60, and 90 min without disinfectant treatment. HuNoV levels were quantified by RNase-RT-qPCR and expressed as log_10_ genome copies/μL. HuNoV levels remained stable during the tested period, with SD values ranging from 0.06 to 0.11 log_10_ genome copies/μL. The dotted horizontal line indicates the limit of quantification (LOQ, 1.60 log_10_ genome copies/μL). Data are presented as mean ± SD.
