# Supplementary material for: Subzero Control of Human Norovirus and Indicator Microorganisms on Frozen Foods Using an Antifreeze–Peracetic Acid Formulation
Source: Viruses. 2026 Aug 1;18(8):846. doi: 10.3390/v18080846 (PMC13517282; doi:10.3390/v18080846)
Supplement: Supplementary file 1 [file viruses-18-00846-s001.zip › viruses-4407489-supplementary.pdf]

Supplementary Material

Supplementary Material Contents

1. Supplementary Table

Table S1. Identification of neutralizing agents for disinfectants

Table S2. Neutralizer validation for HuNoV RT-qPCR

Table S3. Performance of the HuNoV RT-qPCR assay

Table S4. MNV process control recovery after neutralization

Table S5. RT-qPCR inhibition assessment in different food matrix extracts

2. Supplementary Figure

Figure S1. Stability of HuNoV on food matrices at −40 °C

Table S1. Identification of neutralizing agents for disinfectants

Microorganism[a]	Test system[b]	Group[c]	Antifreeze: Average colony forming units of each group[d] (CFU/plate)	Antifreeze: Error rate between groups[e] (%)	Antifreeze-PAA: Average colony forming units of each group (CFU/plate)	Antifreeze-PAA: Error rate between groups (%)
E. coli	microbial suspensions	1	162	4.01	126	4.69
		2	146		121	
		3	158		137	
		4	0		0	
	stainless steel	1	147	3.05	119	5.26
		2	139		105	
		3	151		118	
		4	0		0	
S.aureus	microbial suspensions	1	275	2.87	213	2.10
		2	254		205	
		3	262		217	
		4	0		0	
	stainless steel	1	175	3.71	176	3.35
		2	182		164	
		3	164		178	
		4	0		0	
C.albicans	microbial suspensions	1	175	3.15	185	4.26
		2	183		171	
		3	192		192	
		4	0		0	
	stainless steel	1	132	4.55	156	6.40
		2	145		148	
		3	148		175	
		4	0		0	
A. niger	microbial suspensions	1	50	8.48	53	6.67
		2	53		60	
		3	62		58	
		4	0		0	
	stainless steel	1	55	5.32	56	6.78
		2	50		63	
		3	58		68	
		4	0		0	

Notes: [a] The neutralizer validation assay was performed using *E. coli* and *S. aureus* as the representative bacterium, *C. albicans* as the representative yeast, and *A. niger* spores as the representative mould. [b]All tests were conducted with

PAA at a concentration of 2000 ppm under two test systems, including microbial suspension and stainless steel carrier assays. [c]Four experimental groups were included: 1 neutralizer plus microorganism, 2 disinfectant plus neutralizer plus microorganism, 3 PBS plus microorganism as the growth control, and 4 neutralizer plus PBS plus culture medium as the blank control. [d]Microbial recovery was determined by plate counting and expressed as colony-forming units (CFU/tablet). [e]The consistency of colony recovery among groups was evaluated using the colony count error rate (P_z), calculated as $P_z = \frac{\sum|X-Xi|}{\sum Xi} \times 100\%$, where X represents the mean colony count of the three experimental groups and Xi represents the mean colony count of each individual group.

Table S2. Neutralizer validation for HuNoV RT-qPCR

Test system	Treatment group	Mean ± SD (copies/μL)	Error rate between groupse (%)
Buleberries	Neutralizer + HuNoV	7449.90 ± 538.96	5.15
	(Af-PAA + Neutralizer) + HuNoV	6617.43 ± 301.67	
	PBS + HuNoV	7447.42 ± 360.32	
	PBS + Neutralizer	ND	
Carrots	Neutralizer + HuNoV	7785.68 ± 530.24	4.94
	(Af-PAA + Neutralizer) + HuNoV	7025.09 ± 403.16	
	PBS + HuNoV	7949.54 ± 705.71	
	PBS + Neutralizer	ND	
Chicken breast	Neutralizer + HuNoV	7884.97 ± 579.82	5.58
	(Af-PAA + Neutralizer) + HuNoV	7077.77 ± 134.57	
	PBS + HuNoV	8211.52 ± 917.12	
	PBS + Neutralizer	ND	
Arctic shrimp	Neutralizer + HuNoV	7365.77 ± 643.27	4.28
	(Af-PAA + Neutralizer) + HuNoV	6535.82 ± 606.73	
	PBS + HuNoV	6861.62 ± 1031.09	
	PBS + Neutralizer	ND	

Notes: The consistency among the three HuNoV-containing groups was evaluated using the relative error rate ($P_{(z)}$), calculated as: $P_z = \frac{\sum|X-X_i|}{\sum X_i} \times 100\%$ where X is the overall mean of the three groups and $X_{(i)}$ is the mean value of each individual group. A $P_{(z)}$ value below 10% indicated good consistency,

confirming that neither the neutralizer nor the neutralized Af-PAA interfered with HuNoV RT-qPCR detection. ND, not detected below the limit of detection.

Table S3. Performance characteristics of the HuNoV RT-qPCR assay

Standard curve	Slope	Intercept	R^2	Amplification efficiency (%)
Curve 1	-3.245	36.890	0.999	102.9
Curve 2	-3.290	37.080	0.999	101.4
Curve 3	-3.336	37.777	0.999	99.4
Curve 4	-3.237	36.588	0.999	103.7
Curve 5	-3.416	38.009	0.999	96.20

Note: Values were summarized from five independent HuNV RT-qPCR standard curves. In the standard curve equations, x represents $\log_{10}$ genome copies/μL and y represents the Cq value. Amplification efficiency was calculated using the equation: Efficiency (%) = $[10^{(-1/slope)} - 1] \times 100$.

Table S4. MNV process control recovery after neutralization

Food matrix	MNV input level (genome copies/μL)	MNV detected level (genome copies/μL)	Recovery (%)
Blueberries	1.512×10^3	$(2.01 \pm 0.25) \times 10^2$	13.3
Carrots	1.512×10^3	$(1.87 \pm 0.21) \times 10^2$	12.3
Chicken breast	1.512×10^3	$(2.08 \pm 0.32) \times 10^2$	13.8
Arctic shrimp	1.512×10^3	$(2.04 \pm 0.41) \times 10^2$	13.5

Note: MNV was used as a post-neutralization analytical recovery control. After Af-PAA treatment and neutralization, 100 μL of MNV suspension (1.512×10^3 genome copies/μL) was added to each neutralized sample eluate before concentration and RNA extraction. The final RNA extract volume was 100 μL, giving a theoretical input level of 1.512×10^3 genome copies/μL. MNV was quantified by an MNV-specific RT-qPCR assay targeting the ORF1/ORF2 junction using the forward primer 5′-CACGCCACCGATCTGTTCTG-3′, reverse primer 5′-GCGCTGCGCCATCACTC-3′, and MGB probe 5′-FAM-CGCTTTGGAACAATG-MGBNFQ-3′. Thermal cycling consisted of reverse transcription at 42 °C for 300 s, reverse transcriptase inactivation at 95 °C for 10 min, followed by 45 cycles of 95 °C for 5 s and 60 °C for 20 s. MNV copy numbers were calculated using the corresponding MNV-specific standard curve (TableS4 in Supplementary_Data.xlsx). Detected levels are presented as mean ± SD, and recovery (%) was calculated as the mean detected level divided by the theoretical input level × 100.

Table S5. RT-qPCR inhibition assessment in different food matrix extracts

Food matrix	Cq in nuclease-free water	Cq in matrix extract	ΔCq
Blueberries	22.30 ± 0.17	22.59 ± 0.12	0.30
Carrots	22.33 ± 0.21	22.84 ± 0.18	0.51
Chicken breast	22.30 ± 0.16	22.65 ± 0.17	0.34
Arctic shrimp	22.45 ± 0.18	22.81 ± 0.16	0.36

Note: Note: Values are presented as mean ± SD. ΔCq was calculated as Cq(matrix extract) − Cq(nuclease-free water). Matrix-associated RT-qPCR inhibition was assessed based on the Cq shift between nuclease-free water and matrix extracts. Since all ΔCq values were less than 1 cycle, no apparent RT-qPCR inhibition was observed in the tested food matrix extracts.

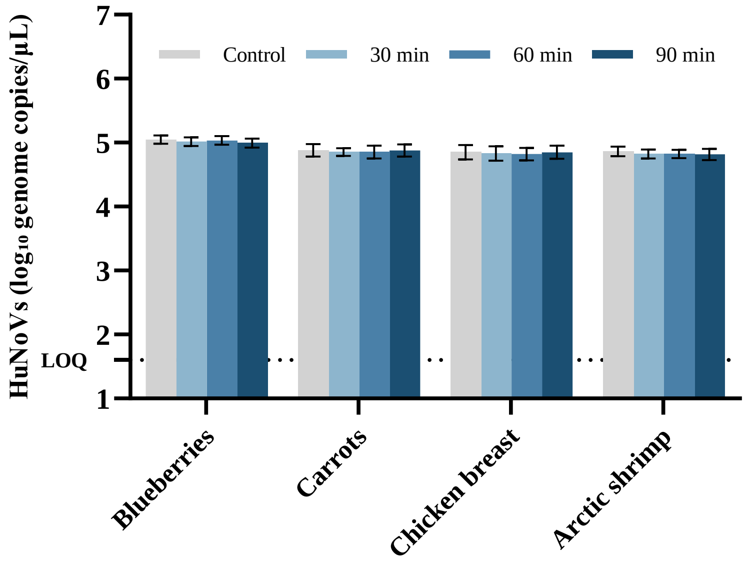

Figure S1. HuNoV-contaminated blueberries, carrots, chicken breast, and Arctic shrimp were held at −40 °C for 30, 60, and 90 min without disinfectant treatment. HuNoV levels were quantified by RNase-RT-qPCR and expressed as $\log_{10}$ genome copies/μL. HuNoV levels remained stable during the tested period, with SD values ranging from 0.06 to 0.11 $\log_{10}$ genome copies/μL. The dotted horizontal line indicates the limit of quantification (LOQ, 1.60 $\log_{10}$ genome copies/μL). Data are presented as mean ± SD.
